# Effects of the COVID-19 school lockdowns on language and math performance of students in elementary schools: implications for educational practice and reducing inequality

**DOI:** 10.1007/s10212-023-00679-4

**Published:** 2023-01-26

**Authors:** Ron Oostdam, Mieke van Diepen, Bonne Zijlstra, Ruben Fukkink

**Affiliations:** 1 Centre for Applied Research in Education (CARE), Faculty of Education, Amsterdam University of Applied Sciences, Wibautstraat 2-4, 1091 GM Amsterdam, The Netherlands; 2 Research Institute of Child Development and Education (RICDE), University of Amsterdam, Amsterdam, The Netherlands

**Keywords:** COVID-19, Primary education, School lockdowns, Urban education, Learning delay, Educational inequality

## Abstract

The current study investigates the effects of the school lockdowns during school years 2019–2020 and 2020–2021 on the achievement scores of primary school students during the COVID-19 pandemic. We analyzed scores for spelling, reading fluency (i.e., decoding speed), reading comprehension, and mathematics from standardized student tracking systems for 5125 students from 26 primary schools in the urban region of The Hague, the Netherlands. Results showed that students in grades 1 through 3 had significant learning delays after the first lockdown. However, results after the second lockdown showed that most students were able to catch up, compared to students from corresponding grades of cohorts before COVID-19. The magnitude of these positive effects was mostly close to the negative effect of the first lockdown. Apparently, during the second lockdown, schools seemed better prepared and able to deliver more effective home schooling and online instruction. The hypothesis that students’ learning from a low SES home environment will suffer most from the school lockdowns could only partly be confirmed. SES effects at the individual level tended to be mitigated by negative effects of SES at the school level, making SES-related differences between schools less profound. The findings of this study offer a broader perspective to evaluate the effects of long-term school closures. Implications for educational practice and issues of inequality between students are discussed.

## Introduction

The past school years 2019–2020 and 2020–2021 have been particularly tough for educational boards, schools, students, and their parents due to the impact of the COVID-19 crisis. In both years, the COVID-19 crisis resulted in a prolonged lockdown of schools and an almost complete switch to distance learning. The pandemic, which effects continue to this day, has impacted students’ cognitive and social-emotional development (Engzell et al., 2020; Gore et al., 2021; Maldonado & De Witte, 2020; Schuurman et al., 2021; Zierer, 2021).

As in many other countries, the Coronavirus crisis has led to elementary schools in the Netherlands being completely closed for about 8 weeks from mid-March 2020 (first lockdown) and a further 8 weeks closure from mid-December 2020 (second lockdown) in the following school year. Only students of parents in so-called vital occupations (e.g., medical health, food supply, and public transport) were allowed to be physically present at school on the days their parents had to work. Additionally, going to school was allowed for students diagnosed as most vulnerable (e.g., students with a problematic home situation, special educational needs, and personality disorders). During the school closures, most students were home-schooled with some online instruction by their teachers, often supported by a parent. However, officially established teaching time was significantly reduced, in many cases to less than 50% (Dutch Inspectorate of Education, 2020, 2021). In the weeks after the lockdowns, all students were allowed to go back to school but in most cases, high-quality education could not yet be realized because not all students returned to school immediately. Some students were absent or difficult to reach for a long time (Dutch Inspectorate of Education, 2020). The added value of the school as a learning environment and meeting place has become increasingly visible due to the pandemic. Most students missed education in the classroom, with their peers and teacher, and despite efforts and guidance of parents at home and the online lessons from teachers, the cognitive and socio-emotional development of many students has slowed down (Dutch Inspectorate of Education, 2020, 2021).

Previous studies into lockdown effects on students’ learning gains have focused on the first lockdown. The first review studies in this domain have reported significant learning gaps for elementary school students, both for the domain of mathematics and reading. Zierer (2021), who summarized findings from five countries (USA, Belgium, the Netherlands, Switzerland, and Germany) in a rapid review, reported a negative effect of school closures on student achievement; the estimated effect size, based on 12 effect sizes from seven studies, was –0.14 standard deviation. There was significant variation between studies. Two studies reported no significant differences for the primary school population, whereas four studies reported a negative change, comparing learning gains from 2020 with results from previous years. Based on their meta-analytic findings of seven studies, Zierer also concluded that primary school students were more affected than secondary school students. The systematic review of Hammerstein and colleagues (2021), which summarized results from 11 studies, also reported a general negative effect of the school lockdown on student’ achievement; the median effect size was − 0.08 standard deviation. Also, this review reported significant heterogeneity of study findings. More recent studies have confirmed the detrimental effects of the school lockdown on students’ learning (see also Gehrer et al., 2022; Schult et al., 2022). Deviant findings are reported in the literature by some authors who did not analyze standardized tests but focused on students’ data from digital learning environments (see Hammerstein et al., 2021), which some schools offer their students to practice language and arithmetic skills. However, it is unclear whether these results can be generalized to students’ regular learning and their achievement on standardized tests (Hammerstein et al., 2021).

The findings from Dutch research which analyzed data from standardized tests fit in with the findings from the reviews. A large cohort study with Dutch data of approximately 350,000 elementary school students showed a learning delay of approximately 3 percentile points or 0.08 standard deviations, after the first lockdown (Engzell et al., 2021). This accumulated learning gap during the period of school closure is equivalent to one-fifth of the learning progress of students in a normal school year.

An investigation into the effects of the first lockdown in the Netherlands mid-March 2020 showed that many students in primary education had significant learning delays in the field of language and math performance. Especially students’ learning of lower-educated parents was affected most (Dutch Inspectorate of Education, 2021). These differences were largest in the lower grades of primary education (see also Zierer, 2021). In addition to the level of education, the income of parents was also an important predictor of learning disadvantage (Dutch Inspectorate of Education, 2021). Considering the educational level of parents, children of high-income parents showed more learning growth than children of parents with low incomes. Finally, migration background played a role, but the influence of this factor was smaller than education or income of parents. Students with a non-Western migration background showed a smaller learning growth in reading comprehension and spelling than their peers without migration background (Dutch Inspectorate of Education, 2021).

The reviews of Hammerstein (2021) and Zierer (2021) have also revealed two moderators of the negative effects of the first school lockdown on students’ learning. First, performance deficits were greater in children from families with a low SES (Hammerstein et al., 2021; Zierer, 2021; see also Engzell et al., 2021; Maldonado & De Witte, 2020). Secondly, specifically younger children were negatively affected by COVID-related school closures (Hammerstein et al., 2021; see also Tomasik et al., 2020). Zierer (2021) adds that students from grades three to five were particularly affected within the elementary school population. A possible explanation for the moderating effect of children’s age is that older students have a higher degree of self-regulation than younger students whose regulatory learning skills are less developed (cf. Bangert-Drowns et al., 1991; Hattie & Timperley, 2007; Schunk & Zimmerman, 2012), but further research is needed to explain differential effects of the school lockdown.

An additional and serious problem, particularly in the urban regions of the Netherlands, is that children of low-educated parents increasingly attend schools that struggle with teacher shortages (Boterman, 2019; Dutch Inspectorate of Education, 2021). Given the possible effects of the school lockdowns, the inequality of opportunity for students in these urban schools may have increased.

### Present study and hypotheses

Complementing the published findings to date, we focus in the present study on the effects of both the first and second lockdowns of elementary schools in the urban part of the Netherlands with a diverse student population, including many disadvantaged students. We analyzed discontinuities in the achievement growth for spelling, reading fluency, reading comprehension, and mathematics of students in grades 1 to 6. In contrast to previous studies that report on effects of the first lockdown, the current study explores the effects of both the first and second periods of lockdown. The main aim is to gain more insight into the effects of both periods of school lockdowns on the learning achievements of different groups of students and in home and school factors that are of influence*.* Insights from the present study aim to contribute to goal-oriented interventions to prevent learning delays as much as possible and to offer equal educational opportunities.

Based on investigations of the Dutch Inspectorate and previous studies on the effects of the first school closure in the Netherlands (Engzell et al., 2020; Schuurman et al., 2021), negative effects are expected on the learning performance of students for the different school subjects. Following Hammerstein et al. (2021) and Zierer (2021), we assume that these negative effects are greater for students from families with a lower SES (*Hypothesis 1*). A related premise is that the effects of the school closures on performance growth of students are related to the proportion of disadvantaged students within schools (*Hypothesis 2*). Especially urban schools with a more disadvantaged student population are less likely to have sufficient resources at their disposal to deal with the difficult situation during the lockdown periods (cf. Andrew et al., 2020; Bayrakdar & Guveli, 2020; Bol, 2020).

We expect more negative effects for younger students (*Hypothesis 3*), based on the reviews of Hammerstein (2021) and Zierer (2021). Especially students in higher grades of primary education may have experienced less adverse consequences from the lockdowns (cf. Hammerstein et al., 2021). Possibly, these students were able to make greater progress at their own learning pace, resulting in increased individual differences between students within classes. School closures may in this way have led to the so-called Matthew effect: “richer” students become richer and “poor” students become poorer (cf. Fisher et al., 2020; Lancker & Parolin, 2020).

Finally, we explore whether the effects of the second school closure have a lesser negative impact because schools were more prepared and geared to provide home education and online instruction, compared to the first lockdown (exploratory *Hypothesis 4*).

## Method

### Participants

For this study, the data of 5125 students from 26 primary schools in the city of The Hague were analyzed. The Hague is an urban region in the Netherlands with large differences between school populations in various neighborhoods; the number of households with children with a low income varies from low (< 7.5%) to high (> 30%) for different districts from The Hague (CBS Factsheet The Hague, 2017). Special education schools were not included in our study.

### Procedure

During the 2020–2021 school year in which the second lockdown took place, fully anonymized data from students were collected in the period February–April 2021. Anonymous transfer of data from the monitoring system of schools for further investigation is permitted and does not require further approval from an ethics committee if information is in no way traceable to individuals.

Due to the time of data transfer, no measurements were available for students at the end of school year 2020–2021. Schools provided the fully anonymized data for students on the tests taken in the middle of school year 2020–2021 and tests taken in the middle and the end of the two preceding years. This implies that for students in grade 1 only data from the 2020–2021 school year were available and for students in grade 2, only data from the year before. Data were available for all other students for 3 successive years. Some schools decided to postpone the measurement at the end of the 2019–2020 school year until after the summer holidays because of the first lockdown mid-March 2020. In the 2020–2021 school year in which the second lockdown took place mid-December 2020, schools sometimes postponed the tests for a few weeks halfway through the school year to the end of March–early April. In those cases, it was nevertheless registered that the tests have been taken at the end or in the middle of the school year according to the standardized test times.

Primary school in the Netherlands starts with 2 years Kindergarten (in Dutch: groepen 1–2) for children aged 4–5. Formal reading and math education begins in grade 1 (in Dutch: groep 3) in August for children who turn 6 years of age between October of the previous year and the following September. To analyze the school achievement of students in grades 1 to 6 schools administer the scores of their students on national curriculum referenced tests for spelling, reading fluency (i.e., decoding speed), reading comprehension, and mathematics. The test scores of students in all six grades were obtained from the participating schools.

The tests are part of the widely used national monitoring and evaluation system developed by CITO, the Dutch National Institute for Educational Measurement (cf. Hollenberg et al., 2017). The system provides a schedule that dictates which tests to take at specific times during the school year (i.e., halfway in January and at the end of June). It enables teachers to monitor their students’ development in relation to both individual and peer development, not only at given times during a school year but also across grades. Tests in the system have good psychometric qualities and are norm referenced. The raw test scores from the different measures of the national monitoring and evaluation system were converted into proficiency scores by means of standardization tables based upon Rasch analysis (cf. Hollenberg et al., 2017). These proficiency scores allow a comparison between different grades or groups. These proficiency scores enable teachers to track the development of a particular student or a class in a particular field, for example reading or mathematics, by displaying the development in a graph.

### Measures

*Spelling* (*S*) is measured in grade 1 by having students writing down words or sentences dictated by the teacher. At the beginning of grade 2, there are two parts: (a) 25 dictated sentences and (b) 25 items for which students must select the sentence with a wrongly spelled word out of four different sentences (Wijs et al., 2006). The time it takes to dictate a word or phrase is related to the teacher’s reading speed and the time students are given to write down the requested word. The manual advises to keep a slow pace and only move on to the next word after all students have finished writing. For the tests with multiple choice questions that are not read out by the teacher, the manual does not state an exact administration time. In general, the duration is a maximum of half an hour per part. The test is taken in class and can be scored objectively by the teacher. The total number of correct responses determines the raw score. Test reliability is above 0.90 (Aarnoutse et al., 2005).

*Fluency of reading* (*FR*) is measured by presenting a word list of the 3-min-test [Drie-Minuten-Toets] (Jongen & Krom, 2009). It is a standardized grade-normed test that comprises three sheets with word lists of increasing difficulty (150 words per sheet, arranged in five rows of 30 words each). Depending on a student’s fluency level, a progressively more difficult card can be used. The test is taken individually by the teacher, a teaching assistant, or an internal supervisor in a quiet place (the hallway or a separate room). Students are instructed to read aloud the 150 words on a sheet as accurately and quickly as possible within 1 min. Wrong pronunciation is not considered an error, but words that are read sound by sound are counted incorrectly, unless the word is pronounced completely afterwards. If a mistake while reading is corrected, the word is also counted correctly. The raw score is the number of words read correctly in 1 min minus the number of words read incorrectly. Reliability is high, ranging from 0.90 to 0.94 (Moelands, Kamphuis & Verhoeven, 2004).

*Reading comprehension* (*RC*) is assessed by requiring students to read text passages silently and to answer inference questions about the texts. There are test booklets for each grade, and a typical test booklet contains between five and ten text passages and 25 multiple choice questions. The class is administered by the teacher in a morning session when the concentration of students is highest. The students receive class instruction on how to take the test. After instruction, the teacher hands out the test booklets that are made individually. Asking questions is not allowed. The test takes approximately 50 min. The raw score is the number of correct answers. Reliability estimates of the test range between 0.88 and 0.93 (Hiddink et al., 2017; Tomesen et al., 2018).

*Mathematics/arithmetic* (*MA*) is tested by measuring students’ problem-solving abilities, both with and without narrative context. The test measures general knowledge of mathematics and arithmetic, and items include problems related to addition, subtraction, multiplication, and division as well as problems concerning the concept of time and the use of money. Students take the test as a class and receive clear instructions beforehand on how to do the exercises. There are test booklets for each grade. The test consists of two parts, a guided and an independent part, and will take approximately 1 h in total. Administration of the guided part means that the teacher reads each math problem aloud and then gives the students sufficient time to find the solution and write it down. The pace of work or problem solving is therefore kept the same for all students in the class. The teacher maintains a pace that gives the average student ample time to follow. When most students have completed a math problem, they move on to the next. The independent part consists of math problems that the students have to solve independently. After a general instruction, the students go to work at their own pace. No further explanation will be given during the test. In principle, each student gets as much time as he/she needs. Those who are ready submit their test sheet to the teacher. The teacher grades the test using clearly prescribed scoring rules. The number of correct responses defines the raw test score. Test reliabilities range between 0.92 and 0.96 (Engelen & Hop, 2017; Engelen et al., 2017; Hop et al., 2016).

*Socio-economic status* (*SES*) of students was estimated based on the digit postal code of their home address consisting of 6 characters (4 digits combined with two letters, e.g., 1001 AB). Statistics Netherlands (CBS) provides three SES indicators based on the first five characters of the postal codes (e.g., 1001 A-), and this unique code is shared by approximately 240 households. The indicators are: (a) the average of the real estate value, (b) the most recent average household income after tax, and (c) the number of people with unemployment or social welfare benefits. Following previous Dutch studies, these three indicators were recoded into a single SES factor score using principal component analysis (Schuurman et al., 2021; Van Leest et al., 2021). Higher scores indicate a higher SES. This factor score has proved a useful marker of SES in previous Dutch studies of elementary schools (Schuurman et al., 2021; Van Leest et al., 2021). Specifically, this SES factor score was a positive predictor of intercept scores in the related study of Schuurman and colleagues, which indicated that students with higher SES generally have higher achievement scores. A preliminary analysis of this SES measure in our study revealed that about half of the variance is within schools (48%), and half of the variance (52%) is between schools.

### Data analyses

Mixed effects models using the package nlme (Pinheiro et al., 2021) in R (R Core Team, 2021) were used to model students’ performance across time for each outcome measure (S, FR, RC, and MA). We applied mixed effects models, taking into account that repeated observations are nested in students, and students are nested in schools. For the repeated observations within students, a completely unrestricted covariance matrix was applied allowing different variances at each timepoint (i.e., heteroscedasticity) and correlations between repeated observations to be freely estimated. At the student-level, this corresponds to a fully multivariate model for the repeated observations (see e.g. Snijders & Bosker, 2011, p. 255–260). Dependence within schools was modeled with a random intercept. The fixed part of each model included the following predictors: the pre-existing difference between cohorts before the lockdowns (L-before), individual SES (SES-I), school-average SES (SES-S), lockdown-1 and lockdown-2 (L1 and L2, indicating whether a lockdown had passed) and two-way interaction effects between the lockdowns and SES (both at the individual and school level). All fixed effects were estimated separately for each grade. In a preliminary analysis, we explored whether gender would moderate findings, but this predictor was often not related to outcomes and, hence, not included in the final models. Residuals were checked for normality and linearity. Importantly, due to the structure of the observed data, the two-way interaction between lockdown-1 and grade estimates the difference in scores of a specific grade, comparing to the same grade in the previous year. The interaction with lockdown-2 estimates the difference comparing to the same grade 2 years ago. We only report the fixed effects estimates, which were of primary interest. Throughout a significance level of 0.01 was used.

## Results

### Descriptives

Tables 1, 2, 3,4 give the proficiency scores for the different cohorts of students, broken down by grade and test administration at the middle or the end of the school year. Cohort 1 contains students that were currently in grade 1 in the school year 2020–2021 (the moment of data collection); cohort 2 contains students that were in grade 2 in school year 2020–2021, and so on. Numbers in italics indicate administration after the first lockdown mid-March 2020 of the 2019–2020 school year; numbers in bold indicate administration after the second lockdown mid-December 2020 of the 2020–2021 school year. In the remaining cells, the scores in the two previous school years are given of students within a cohort (except for students in cohorts 1–2 because no scores were available for the preceding years). The scores in previous years without a lockdown are used as reference values in the analyses.

**Table 1:** Achievement scores (*SD* in brackets) for spelling (S) per cohort, broken down by grade and administration in the middle and the end of the school year (scores after the first lockdown are in italics, those after the second in bold)

S	Cohort 1 N = 765	Cohort 2 N = 852	Cohort 3 N = 862	Cohort 4 N = 864	Cohort 5 N = 802	Cohort 6 N = 684
*Grade 1 middle*	**148 (55)**	159 (57)	160 (54)			
*Grade 1 end*		*199 (49)*	212 (44)			
*Grade 2 middle*		**249 (47)**	247 (46)	246 (42)		
*Grade 2 end*			*277 (47)*	278 (43)		
*Grade 3 middle*			**300 (43)**	298 (44)	302 (43)	
*Grade 3 end*				*320 (45)*	325 (43)	
*Grade 4 middle*				**322 (37)**	326 (38)	325 (41)
*Grade 4 end*					*344 (37)*	346 (39)
*Grade 5 middle*					**357 (34)**	357 (33)
*Grade 5 end*						*368 (34)*
*Grade 6 middle*						**379 (37)**

Cohort 1 contains students that were in grade 1 in the school year 2020–2021 (the moment of data collection); cohort 2 contains students that were in grade 2 in school year 2020–2021, etc. In addition to the scores after the first and second lockdowns, the other cells contain the scores of students within a cohort in previous school years, which are used as a reference value in the analyses

**Table 2:** Achievement scores (*SD* in brackets) for fluency of reading (FR) per cohort, broken down by grade and administration in the middle and the end of the school year (scores after the first lockdown are in italics, those after the second in bold)

FR	Cohort 1 N = 0	Cohort 2 N = 725	Cohort 3 N = 744	Cohort 4 N = 741	Cohort 5 N = 658	Cohort 6 N = 602
*Grade 1 middle*	**-**	18 (13)	19 (13)			
*Grade 1 end*		*32 (18)*	34 (17)			
*Grade 2 middle*		**50 (19)**	46 (23)	48 (19)		
*Grade 2 end*			*57 (20)*	58 (19)		
*Grade 3 middle*			**67 (19)**	68 (20)	70 (18)	
*Grade 3 end*				*74 (19)*	77 (18)	
*Grade 4 middle*				**81 (19)**	84 (18)	84 (17)
*Grade 4 end*					*86 (19)*	89 (17)
*Grade 5 middle*					**92 (17)**	93 (17)
*Grade 5 end*						*98 (16)*
*Grade 6 middle*						**103 (17)**

**Table 3:** Achievement scores (*SD* in brackets) for reading comprehension (RC) per cohort, broken down by grade and administration in the middle and the end of the school year (scores after the first lockdown are in italics, those after the second in bold)

RC	Cohort 1 N = 0	Cohort 2 N = 764	Cohort 3 N = 842	Cohort 4 N = 824	Cohort 5 N = 756	Cohort 6 N = 647
*Grade 1 middle*	**-**	-	-			
*Grade 1 end*		*114 (30)*	118 (28)			
*Grade 2 middle*		**132 (30)**	133 (28)	134 (28)		
*Grade 2 end*			*138 (28)*	141 (27)		
*Grade 3 middle*			**149 (29)**	152 (27)	152 (28)	
*Grade 3 end*				*153 (27)*	156 (28)	
*Grade 4 middle*				**165 (27)**	168 (26)	171 (28)
*Grade 4 end*					*176 (28)*	181 (29)
*Grade 5 middle*					**182 (30)**	191 (29)
*Grade 5 end*						*196 (28)*
*Grade 6 middle*						**209 (33)**

**Table 4:** Achievement scores (*SD* in brackets) for mathematic/arithmetic (MA) per cohort, broken down by grade and administration in the middle and the end of the school year (scores after the first lockdown are in italics, those after the second in bold)

MA	Cohort 1 N = 798	Cohort 2 N = 856	Cohort 3 N = 876	Cohort 4 N = 883	Cohort 5 N = 806	Cohort 6 N = 734
*Grade 1 middle*	**111 (32)**	115 (36)	116 (32)			
*Grade 1 end*		*138 (29****)***	144 (29)			
*Grade 2 middle*		**165 (31)**	163 (31)	166 (31)		
*Grade 2 end*			*183 (30)*	188 (28)		
*Grade 3 middle*			**203 (31)**	203 (29)	204 (28)	
*Grade 3 end*				*213 (30)*	214 (28)	
*Grade 4 middle*				**226 (27)**	226 (28)	230 (28)
*Grade 4 end*					*237 (29)*	244 (28)
*Grade 5 middle*					**251 (30)**	252 (28)
*Grade 5 end*						*262 (28)*
*Grade 6 middle*						**273 (31)**

Effects for the *first* lockdown were tested by comparing a cohort with the adjacent cohort from the previous year (see Analysis, “Method” section). For example, the spelling scores of students in grade 1 from cohort 2 immediately after the first lockdown (*M* = 199; *SD* = 49) are compared with the scores of the students who were in grade 1 in cohort 3 (*M* = 212; *SD* = 44). When comparing these scores, a learning delay is visible (*M* = 199 versus *M* = 212). Thus, we compared the scores in italics across the same grades in adjacent cohorts.

Effects for the *second* lockdown were tested by comparing scores from a cohort with the cohort from 2 years earlier; so we compared the scores in bold for the same grades for cohorts 2 years earlier. For example, compared to the scores of students from cohort 3 in grade 1 (*M* = 160; *SD* = 54), the spelling scores of students in grade 1 of cohort 1 (*M* = 148; *SD* = 55) show a delay (160–148 = 12).

At the same time, the table shows that such a learning delay is not clearly visible for the other grades after the first lockdown. After the second lockdown, the same pattern can be observed. Just like after the first lockdown, the scores of students in all cohorts after the second lockdown (numbers in bold) show no major differences with the scores of students in corresponding grades in school years without a lockdown. For example, comparing the average mid score for spelling of the grade 2 students in cohort 2 in the 2020–2021 school year (*M* = 249; *SD* = 47) with the average grade 2 score of the current grade 3 students in cohort 3 (*M* = 247; *SD* = 46), and the current grade 4 students in cohort 4 (*M* = 246; *SD* = 42) hardly shows any differences.

A close inspection of Table 2 shows that the mean scores for FR only show minor differences in achievement scores across cohorts. This pattern is the same after both lockdown periods. The scores for reading comprehension (see Table 3), on the other hand, mainly show negative effects of the first lockdown. A horizontal comparison of the scores (numbers in italics) between the cohorts shows a fairly consistent pattern of learning delays. At the same time, it is visible that this pattern also occurs after the second lockdown but apparently not to the same degree.

Further analyses of the MA scores (see Table 4) reveal negative effects after the first lockdown (numbers in italics) for grade 1 of cohort 2 (*M* = 138; *SD* = 29) and grade 3 of cohort 3 (*M* = 183; *SD* = 30) and grade 4 of cohort 5 (*M* = 237; *SD* = 29), compared to the corresponding grade scores of students in school years without a lockdown period. There appears to be no negative effect of the second lockdown.

Inspection of Tables 1, 2, 3,4 shows that the means of the various tests show small differences in achievement scores across cohorts. The negative effects of the first lockdown generally seem to be somewhat greater than those of the second lockdown.

### Testing research hypotheses

Tables 5, 6, and 7 report the effects for three learning outcomes, and they include each three effects: a baseline before the lockdown (before L), the effect of the first lockdown (L1), and the effect of the second lockdown (L2). In addition, we report the effect of socio-economic status at both individual student level (SES-I) and school level (SES-S). To explore possible differential lock down effects related to individual and/or school SES, we also included interaction effects of SES with the effect of the first and second lockdowns.

**Table 5:** Fixed effects for spelling (S), broken down per grade before lockdown (before L) and after the first (L1) and second (L2) lockdowns, without and with SES at individual (I) and school (S) levels

	*Grade 2*		*Grade 3*		*Grade 4*		*Grade 5*	
	Estimate	*p*-value	Estimate	*p*-value	Estimate	*p*-value	Estimate	*p*-value
*S Before L* *S SES-I* *S SES-S* *S L1* *S L1* × *SES-I* *S L1* × *SES-S* *S L2* *S L2* × *SES-I* *S L2* × *SES-S*	2.893 2.366 9.033 − 13.861 1.908 − 10.207 12.916 − 0.544 − 3.532	0.248 0.416 0.026 < 0.001 0.387 < 0.001 < 0.001 0.804 0.229	− 2.831 − 0.070 1.932 − 1.844 1.296 − 3.607 1.534 2.666 2.159	0.187 0.974 0.547 0.254 0.373 0.082 0.533 0.097 0.342	− 2.430 − 0.419 1.190 − 1.102 1.894 0.734 2.171 − 1.714 2.322	0.236 0.824 0.691 0.454 0.152 0.708 0.331 0.230 0.273	2.534 4.499 − 6.447 − 2.807 0.036 2.044 3.167 − 0.099 − 4.078	0.168 0.012 0.017 0.032 0.977 0.218 0.019 0.939 0.020

**Table 6:** Fixed effects on fluency of reading (FR) and reading comprehension (RC), broken down per grade before (before L) and after the first (L1) and second (L2) lockdowns, without and with SES at individual (I) and school (S) levels

	*Grade 2*		*Grade 3*		*Grade 4*		*Grade 5*	
	Estimate	*p*-value	Estimate	*p*-value	Estimate	*p*-value	Estimate	*p*-value
*FR Before L* *FR SES-I* *FR SES-S* *FR L1* *FR L1* × *SES-I* *FR L1* × *SES-S* *FR L2* *FR L2* × *SES-I* *FR L2* × *SES-S*	− 0.721 − 0.289 2.398 − 1.065 0.743 − 2.216 4.726 0.199 2.040	0.324 0.725 0.060 0.034 0.204 0.005 < 0.001 0.734 0.010	0.856 − 0.903 3.150 − 0.973 − 0.298 0.404 − 1.839 0.023 2.147	0.393 0.232 0.012 0.041 0.521 0.545 0.076 0.964 0.003	− 2.571 0.144 1.775 0.912 − 0.027 0.412 − 1.771 0.067 3.392	0.008 0.883 0.270 0.049 0.952 0.537 0.080 0.896 < 0.001	− 0.597 1.119 − 0.100 − 1.619 0.373 − 0.032 0.241 − 0.857 1.547	0.531 0.283 0.950 < 0.001 0.433 0.961 0.594 0.095 0.029
*RC Before L* *RC SES-I* *RC SES-S* *RC L1* *RC L1* × *SES-I* *RC L1* × *SES-S* *RC L2* *RC L2* × *SES-I* *RC L2* × *SES-S*	- - - − 2.747 2.498 − 3.679 1.376 0.909 − 2.495	- - - 0.070 0.090 0.111 0.412 0.447 0.116	-2.453 3.780 − 5.752 − 2.729 − 0.583 1.227 2.860 0.727 1.515	0.081 0.003 0.007 0.015 0.553 0.374 0.062 0.474 0.289	1.086 2.048 − 4.081 − 2.714 2.546 − 0.883 − 2.921 − 1.148 3.145	0.414 0.078 0.052 0.008 0.003 0.486 0.051 0.257 0.032	1.086 3.556 − 3.443 − 3.225 − 0.212 1.154 − 0.675 1.764 − 1.617	0.414 0.002 0.082 0.002 0.819 0.357 0.534 0.116 0.285

**Table 7:** Fixed effects on mathematics/arithmetic (MA), broken down per grade before (before L) and after the first (L1) and second (L2) lockdowns, without and with SES at individual (I) and school (S) levels

	Grade 2		Grade 3		Grade 4		Grade 5	
	Estimate	*p*-value	Estimate	*p*-value	Estimate	*p*-value	Estimate	*p*-value
*MA Before L* *MA SES-I* *MA SES-S* *MA L1* *MA L1* × *SES-I* *MA L1* × *SES-S* *MA L2* *MA L2* × *SES-I* *MA L2* × *SES-S*	− 0.301 4.094 0.736 − 4.718 − 0.169 − 3.773 3.667 0.335 − 3.632	0.832 0.012 0.754 < 0.001 0.862 0.004 0.019 0.752 0.011	− 4.639 4.063 − 6.549 − 2.816 0.833 − 1.657 6.084 0.053 1.204	0.001 0.003 0.002 0.002 0.308 0.154 < 0.001 0.958 0.403	− 0.456 4.121 − 8.733 − 0.905 0.497 2.306 0.348 − 0.230 − 0.066	0.740 0.001 < 0.001 0.264 0.492 0.031 0.808 0.786 0.958	− 1.577 4.674 − 8.229 − 3.635 1.357 − 1.860 4.700 1.479 0.384	0.208 < 0.001 < 0.001 < 0.001 0.070 0.068 < 0.001 0.112 0.763

Table 5 shows that only students in grade 2 have a significantly lower score in their spelling performance after the first lockdown. After the first lockdown, they score about 14 units lower (i.e., − 13.86, *p* < 0.001). This effect takes into account the estimated pre-existing difference of 2.893 (S before L, comparing grade 2 before the first lockdown to the preceding cohort, 1 year earlier), which is not significant. The first lockdown effect is not related to individual SES (1.908, n.s.), but there is a significant effect of school average for SES (− 10.207, *p* < 0.001). Schools with relatively many students with a high SES performed thus worse after the first lockdown than schools with students with a lower SES on average in grade 2. Schools with many students with a high SES can actually drop more in terms of learning delay than schools with many students with a low SES because these students already score relatively low on average.

Table 5 also shows that, after the initial relapse of the first lockdown (− 13.861), grade 2 students made significant progress after the second lockdown (+ 12.916) and caught up again with their peers from previous cohorts. So, the learning delay in grade 2 incurred during the first lockdown was no longer apparent after the second lockdown. This positive effect of the second lockdown was unrelated to SES at either individual or school level, as the non-significant interaction effects indicate.

Finally, no negative effects of either school lockdown 1 or 2 related to spelling were found in all other grades.

Table 6 shows that there was a significant negative effect for FR after the first lockdown in grade 5 (− 1.619). After the second lockdown, there was a significant learning advantage for FR in grade 2 (4.726). In grade 2, there was also a negative interaction effect between SES at school level and lockdown 1, followed by a similar positive interaction effect after lockdown 2. In grades 3 and 4, there was a positive interaction effect between SES at school level and lockdown 2 as well. These positive effects favored higher SES schools.

As far as reading comprehension is concerned, there were significant declines in grades 4 and 5 after the first lockdown, but in the other grades, no significant effects occurred. There was a significant positive effect of individual level SES in grade 3, contrasted by a significant negative effect of SES at school level. In grade 5, there was a significant positive effect of SES at the individual level. In grade 4, there was a significant positive interaction between lockdown 1 and individual level. All of the significant individual effects of SES favored higher SES individuals, while all of the significant school level effects favored lower SES schools.

The scores for mathematics/arithmetic (see Table 7) showed a significant decline for grades 2, 3, and 5 after the first lockdown, combined with a significant increase after the second lockdown for grades 3 and 5. There were significant positive effects of SES at the individual level in all grades, contrasted by a negative effect at school level in grades 3, 4, and 5. A negative interaction effect with school-level SES after the first lockdown was observed in grade 2. Similar to reading comprehension, again, all significant individual-level SES effects favor higher SES pupils, while all school-level SES effects favor lower SES schools.

In sum, when a significant effect occurs, there is a general pattern for our outcome measures with a negative effect of the first lockdown (L1), as expected. In addition, we see positive recovery effects after the second lockdown (L2). The magnitude of these positive effects was mostly close to the negative effect of the first lockdown. For spelling, the combination of both effects was observed for the youngest children in grade 2. For mathematics, both effects were combined in grades 3 and 5. For reading comprehension and mathematics, significant effects of SES, either as main effect or in interaction with lockdown, at the individual level, all are positive, while the effects of SES at the school level are negative. It is important to note that the negative effects at the school level (1) do not reflect a decline but a less strong increase over time and (2) should be interpreted taking into account the individual level effect of SES; these tend to be opposing. For spelling and fluency of reading the effects of SES were more heterogeneous.

## Discussion and conclusion

The results of this study, in which the school performance of 5125 students from 26 primary schools in the Netherlands were analyzed on standardized tests from a national monitor system, provides the first insight into the combined effects of both the first and second lockdowns during the school year 2019–2020 (from mid-March to mid-May 2020) and 2020–2021 (from mid-December 2020 to mid-February 2021).

The previously reported, negative effects of the first school closure in the Netherlands (Dutch Inspectorate of Education, 2021; Engzell et al., 2020; Schuurman et al., 2021) were largely confirmed by the current study. During the first lockdown, students in grades 1–3 had significant learning delays across achievement tests. At the same time, there were learning delays for students in the higher grades, but to a limited extent. This result for elder students corresponds to the study of Gore et al. (2021). Drawing on data from more than 4800 year 3 and 4 students from 113 government schools, no significant differences were found between 2019 and 2020 in student achievement growth, as measured by progressive achievement tests in mathematics or reading. However, the statistically significant learning delays were relatively modest, and the effect sizes are small. This finding is in line with previous studies, concerning the effects of the recent school lockdowns (see Engzell et al., 2020; Hammerstein et al., 2021).

During the second lockdown, students from all grades were catching up in our study. On all achievement tests taken after the second lockdown, students showed, on average, no longer significant differences with students from the reference year without a lockdown at the same schools. Just as in case of the first lockdown, the test results after the second lockdown showed no significant learning delays for students in higher grades either.

While teachers, educators, and policy makers generally focus on negative effects of the school closures on students with lower socio-economic home situations (e.g., Bol, 2020; Kuhfeld et al., 2020), this concern is not reflected by the results of this study (see also Schuurman et al., 2021 for a similar outcome). Of course, this does not mean that there are no effects of SES on an individual level. Since a long time, studies have regularly confirmed that students with a lower individual SES score lower on average on achievement tests than students with a higher individual SES (e.g., Bosker, Mulder & Glas, 2001; Dutch Inspectorate of Education, 2009). The results of this study did confirm for some outcomes that these differences have significantly been increased due to a specific lockdown effect. Our first hypothesis that students’ learning from a low SES home environment will suffer most from the school lockdowns can therefore be partly confirmed. These effects, however, tended to be mitigated by negative effects of SES at the school level making SES-related differences between schools less profound. Therefore, our second research hypothesis that the negative effects of the school closures on students’ achievement scores is related to the proportion of disadvantaged students within schools could not be confirmed.

Furthermore, our results provide some support for our third hypothesis related to the negative effects of the school closures for younger students. This is in line with the review study of Hammerstein et al. (2021) and Zierer (2021), indicating that specifically the student achievement of younger children was negatively affected by the COVID-19-related school closures.

The positive development during the second lockdown seems to confirm our fourth and final hypothesis which predicted fewer negative effects of the second lockdown compared to the first lockdown. Possibly, schools were better prepared this time and able to deliver more effective home schooling and online instruction during the second lockdown. In addition, during the first lockdown, the younger students in our study showed adverse effects, but these effects disappeared during the second lockdown. A decisive factor during the second lockdown may be that the schools exclusively focused on monitoring language and math test performance within the broader school curriculum and paid less attention to other curricular topics.

### Strengths and limitations

Some limitations of the current study should be discussed. For example, it was not possible to examine the effects of the lockdowns on the progression of students from grade 6 to secondary education. As a result, it remains unclear to what extent the school lockdown(s) may have had a negative impact on referrals to secondary education. Furthermore, this study focused on the effects of the lockdown periods on cognitive achievements. The broad development regarding socialization and personalization of students (Biesta, 2015) was beyond the scope of our study, while the school closures could very well have a much greater negative impact on these aspects. Related to this, a study in which quantitative data of achievement scores are analyzed in combination with more qualitative data of what happens at school and home may provide more insight in the learning outcomes and possible loss of individual students.

Regarding the percentage of explained variance, which indicates how well school factors predict students’ reading and math performance, different figures are mentioned. Sometimes the figures of 40–50% is mentioned in connection with the home and family factors (cf. Dutch Inspectorate of Education, 2006), but according to the study of Marzano schools on average explain only 20% of the variance of student scores (Marzano, 2007, 12). This underlines the importance of a broad approach in analyzing student performance in relation to environmental and background characteristics, individual attributes, and the learning environment at school (cf. Bronfenbrenner & Morris, 1998). In the specific context of school lockdowns, future research may provide more insight into the learning conditions at home, support of siblings, and/or other support in the (extended) family.

A strength of the study is that it covered many schools that are estimated to provide a representative picture of elementary schools in an urban environment with students from different cultural backgrounds. In addition, this study not only analyzed effects of the first, but had an integrated perspective on effects of the first and second lockdowns on achievement tests administered in all grades of elementary education. This integrated analysis adds new insights to the very first COVID-19 studies in relation to the adaptability of schools to respond to this exceptional and unforeseen situation of school lockdowns.

### Conclusion

Based on the findings of this study, the effects of the COVID-19 pandemic on the long-term closures of schools can be evaluated from a broader perspective. Although studies into the effects of the first lockdown period discussed learning disadvantages and an increased chance of inequality of opportunity, the present study showed that there is a significant catch-up at student level during and after the second lockdown. This is remarkable because research by the Dutch Inspectorate showed that the effective teaching time was sometimes reduced by half during the lockdown periods (Dutch Inspectorate of Education, 2020, 2021). Apparently, the total effective teaching time to be spent is less important for catching up. Speaking of a COVID-19 school generation with students who are hampered in their future school careers due to the negative effects of the school lockdowns, seems therefore premature when it comes to students’ cognitive performance level. If there is a serious impediment, it may be more related to problems surrounding the broader social-emotional development of students of which the educational practice (school boards and teachers) is increasingly signaling (Dutch Inspectorate of Education, 2020, 2021).

Currently, as in other countries, the Dutch government provides schools with additional financial resources to reduce or eliminate learning delays because of the pandemic and to push back inequality of opportunity. The results of this study, however, raises the question whether the focus of such a policy measure should be primarily on reducing differences between students’ cognitive achievement levels on a generic school level. Schools may be better off investing in the learning and broader development of students with special needs in a targeted approach.

## Data Availability

Anonymized data files are available and can be requested.
